# Quantitative Approach to Fragmented QRS in Arrhythmogenic Cardiomyopathy: From Disease towards Asymptomatic Carriers of Pathogenic Variants

**DOI:** 10.3390/jcm9020545

**Published:** 2020-02-17

**Authors:** Rob W. Roudijk, Laurens P. Bosman, Jeroen F. van der Heijden, Jacques M. T. de Bakker, Richard N. W. Hauer, J. Peter van Tintelen, Folkert W. Asselbergs, Anneline S. J. M. te Riele, Peter Loh

**Affiliations:** 1 Department of Cardiology, Division Heart & Lungs, University Medical Center Utrecht, Utrecht University, 3508 GA Utrecht, The Netherlands; r.w.roudijk@umcutrecht.nl (R.W.R.); L.P.Bosman-4@umcutrecht.nl (L.P.B.); J.F.vanderHeijden-2@umcutrecht.nl (J.F.v.d.H.); R.N.W.Hauer@umcutrecht.nl (R.N.W.H.); F.W.Asselbergs@umcutrecht.nl (F.W.A.); ariele@umcutrecht.nl (A.S.J.M.t.R.); 2 Netherlands Heart Institute, 3511 EP Utrecht, The Netherlands; J.P.vanTintelen-3@umcutrecht.nl; 3 Department of Clinical and Experimental Cardiology, Amsterdam University Medical Center, 1105 AZ Amsterdam, The Netherlands; j.m.debakker@amsterdamumc.nl; 4 Department of Genetics, University Medical Center Utrecht, Utrecht University, 3508 GA Utrecht, The Netherlands; 5 Institute of Cardiovascular Science, Faculty of Population Health Sciences, University College London, London WC1E, UK; 6 Health Data Research UK and Institute of Health Informatics, University College London, London WC1E, UK

**Keywords:** electrocardiography, fragmented QRS, fQRS, ventricular arrhythmia, sudden cardiac death, arrhythmogenic cardiomyopathy, inherited cardiomyopathies, genetics

## Abstract

Fragmented QRS complexes (fQRS) are common in patients with arrhythmogenic cardiomyopathy (ACM). A new method of fQRS quantification may aid early disease detection in pathogenic variant carriers and assessment of prognosis in patients with early stage ACM. Patients with definite ACM (*n* = 221, 66%), carriers of a pathogenic ACM-associated variant without a definite ACM diagnosis (*n* = 57, 17%) and control subjects (*n* = 58, 17%) were included. Quantitative fQRS (Q-fQRS) was defined as the total amount of deflections in the QRS complex in all 12 electrocardiography (ECG) leads. Q-fQRS was scored by a single observer and reproducibility was determined by three independent observers. Q-fQRS count was feasible with acceptable intra- and inter-observer agreement. Q-fQRS count is significantly higher in patients with definite ACM (54 ± 15) and pathogenic variant carriers (55 ± 10) compared to controls (35 ± 5) (*p* < 0.001). In patients with ACM, Q-fQRS was not associated with sustained ventricular arrhythmia (*p* = 0.701) at baseline or during follow-up (*p* = 0.335). Both definite ACM patients and pathogenic variant carriers not fulfilling ACM diagnosis have a higher Q-fQRS than controls. This may indicate that increased Q-fQRS is an early sign of disease penetrance. In concealed and early stages of ACM the role of Q-fQRS for risk stratification is limited.

## 1. Introduction

Sudden cardiac death (SCD) may be the first manifestation of the disease in patients with arrhythmogenic cardiomyopathy (ACM) and asymptomatic carriers of pathogenic ACM-associated variants [1]. The most frequently detected and well described subcategory of ACM is arrhythmogenic right ventricular cardiomyopathy (ARVC), in which abnormalities are predominantly (but not exclusively) found in the right ventricle (RV). Routine 12-lead electrocardiography (ECG) has a major role in the 2010 Task Force Criteria (TFC) for ARVC diagnosis [2]. Indeed, recent studies have shown that fragmentation of QRS complexes is common in ACM and reflects electrical dyssynchrony due to anisotropic activation pathways, load mismatch, cardiomyocyte disconnection and altered tissue architecture by fibrofatty alteration [3]. In ischemic heart disease, fragmented QRS complexes (fQRS) are associated with myocardial scar burden and sustained ventricular arrhythmias (SVA) [4,5,6,7]. Likewise, prior studies suggested that fQRS reflects severity of the disease and appearance of SVA in ACM cohorts [8,9]. However, the significance of fQRS for early disease detection and prognosis remains uncertain [10,11].

Outcome of previous studies may have been affected by different patient categories, limited molecular-genetic testing, inclusion of patients taking anti-arrhythmic drugs (AAD) and the use of various filter settings of the ECG [8,9]. Moreover, all usual but slightly varying fQRS measurement methodologies are prone to subjectivity. With these limitations, fQRS remains a qualitative and operator-dependent ECG characteristic with poor reproducibility between studies [4,11,12]. 

In this study we aimed to (1) reproducibly quantify fQRS in patients with definite ACM and in pathogenic variant carriers without definite ACM diagnosis and (2) assess quantitative fQRS (Q-fQRS) as diagnostic tool and risk marker.

## 2. Experimental Section

### 2.1. Study Population

Patients diagnosed with definite ACM according to the 2010 TFC (at least two major TFC, one major and two minor TFC or four minor TFC) and both symptomatic and asymptomatic carriers of a pathogenic ACM-associated variant without definite ACM diagnosis (either one major TFC, one major and one minor TFC) were included between 1991 and 2019 in the multicenter Netherlands ACM Registry [2,13]. Patients with definite ACM were divided for subgroup analysis in groups with and without prior history of SVA episodes. Pathogenic variant carriers without definite ACM were deemed symptomatic when they experienced palpitations, chest pain or cardiac syncope. Control subjects were included from the non-athlete control group of a previous study which evaluated the cardiovascular characteristics of athletes [14]. For patients with definite ACM the age at diagnosis was used for analysis. For pathogenic variant carriers and controls the age at the moment of ECG recording was used. All control subjects were evaluated using ECG, echocardiography and cardiac magnetic resonance imaging (CMR). Patients with ACM and pathogenic variant carriers underwent clinical evaluation by their treating physician. The study was approved by the local institutional ethics review board.

### 2.2. Data Collection

**Figure 1:**
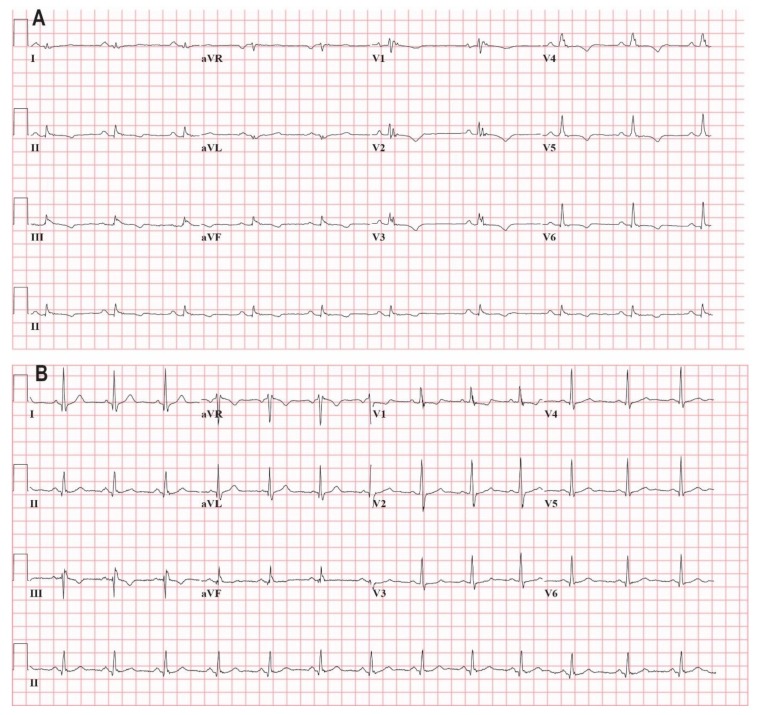
(**A**): Resting electrocardiography (ECG) (25 mm/s, 10 mm/mV, 150 Hz) of a 43 year old female patient with ACM and a pathogenic plakophilin 2 (*PKP2*) variant (c.2368T > C p.Cys796Arg)) who presented with recurrent sustained ventricular tachycardia (VT). She underwent unsuccessful VT ablation and afterwards an implantable cardioverter defibrillator (ICD) implantation. During follow-up, she developed therapy resistant heart failure due to RV failure and underwent heart transplantation. Her resting ECG without antiarrhythmic drugs showed sinus bradycardia, prolonged QRS duration (160 ms) due to intraventricular conduction disorder, prolonged terminal activation duration (TAD, 110 ms) and T wave inversion in leads II, III, aVF, V1–V6. There is marked fragmentation of the QRS complexes in the inferior- and precordial leads. (**B**): Resting ECG (25 mm/s, 10 mm/mV, 150 Hz) of a 28 year old male who is a carrier of a pathogenic *PKP2* variant (c.379C > T p.(Gln133 *)) and a variant of unknown significance in *PKP2* (c.2615C > T p.(Thr872Ile)). He was included in the pathogenic variant carrier group. The patient was referred for family cascade screening and is asymptomatic. Cardiac MRI showed a normal RVEF (49%) and LVEF (63%), normal RV volumes, no focal wall motion abnormalities and subtle atypical late gadolinium enhancement midmyocardial in the RV. Holter monitoring and exercise testing were normal. His resting ECG showed counter clockwise rotation, sinus rhythm, normal QRS duration (108 ms) and T wave inversion in III, aVR and V1. The QRS complexes have marked fragmentation in the inferior leads, aVL, V1 and V2.

Routine 12-lead ECGs (GE Healthcare MAC5500, Chicago, IL, USA) were performed with 25 mm/s paper speed, 10 mm/mV resolution, 150 Hz low pass filter and 400 Hz sampling frequency. ECGs recorded during atrial fibrillation or with low pass filter settings < 150 Hz were excluded from analysis. All ECGs were stored as PDF files and reviewed at 5× magnification. Examples of ECGs are depicted in Figure 1. Included ECGs were recorded at presentation, the moment of ACM diagnosis, or within one year after diagnosis. Patients were excluded from the study if Vaughan Williams class I, III or IV AAD were used during ECG acquisition [15], the use of beta-blockers (class II) was allowed. Clinical data were retrospectively collected according to the Netherlands ACM Registry protocol and included clinical history, molecular-genetic testing, ECG, 48 h Holter recording, echocardiography and CMR [13]. 

### 2.3. Total Q-fQRS

**Figure 2:**
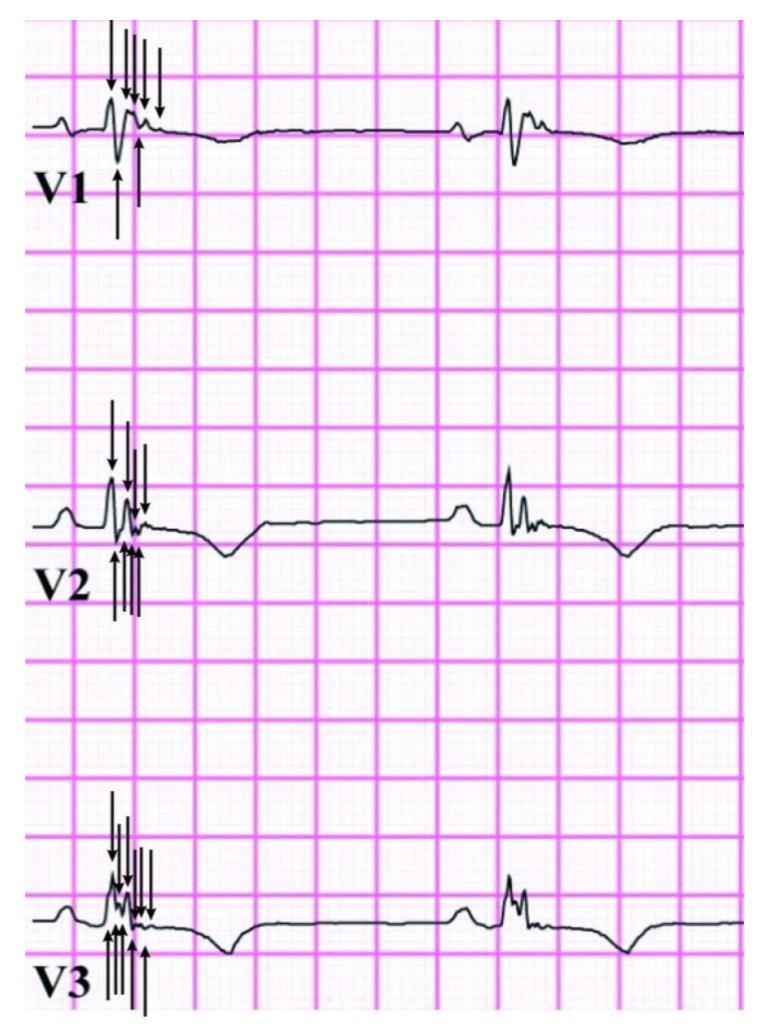
This figure shows recordings of leads V1–V3 from a patient with arrhythmogenic cardiomyopathy. All shown QRS complexes have fQRS according to the definition by Das [4]. The quantitative fQRS (Q-fQRS) counting is indicated by black arrows. Each arrow represents a positive or negative deflection counted as fractionated signal and was reproducible between the first and second QRS complex of each lead. Lead V1, V2, and V3 have a Q-fQRS count being 7, 8, and 11, respectively. The Q-fQRS count for leads V1–V3 in this figure add up to 26 (for the total Q-fQRS is summation of deflections in all 12 ECG leads required, these leads are not shown).

To improve reproducibility, Q-fQRS was designed to overcome the operator-dependent assessment of the qualitative methods used in prior literature [5]. The Q-fQRS method quantifies the total amount of fragmentation in all 12 routine ECG leads together. The summation of absolute numbers of positive and negative deflection points in each first QRS complex of each ECG lead were labeled as a fQRS count value (Figure 2). The first deviation from the iso-electric line and the last transition from the last deflection to the iso-electric line were excluded. To minimize the count of mechanical artifacts, these deflections had to be reproducible between the first and second QRS complex of each lead. Non-reproducible deflections between consecutive QRS complexes were deemed mechanical or electrical artifacts and were not added to the Q-fQRS count value. All ECGs were scored by a single trained observer, blinded for the phenotype and arrhythmia outcome. Reproducibility was determined comparing a random sample of 30 ECGs which were scored by three independent and blinded observers. Total Q-fQRS was compared with Terminal Activation Duration (TAD) as alternative depolarization parameter. TAD was defined as the interval between the nadir of the S wave and the end of all depolarization deflections in ECG leads V1-3 [16].

### 2.4. Follow-up

ACM patients and pathogenic variant carriers were followed at outpatient clinic visits at least once a year and once every two years, respectively. Each visit included at least clinical history, 12-lead ECG recording, and echocardiography. Total Q-fQRS at baseline and SVA during follow-up were used as prognostic markers in ACM patients without SVA at baseline and in the pathogenic variant carriers. The primary outcome was occurrence of SVA during follow-up, which was defined as a combined endpoint of SCD, resuscitated cardiac arrest (SCA), sustained ventricular tachycardia (VT), ventricular fibrillation (VF) and appropriate implantable cardioverter defibrillator (ICD) therapy [17]. 

### 2.5. Statistical Methods

Statistical analysis was performed in RStudio version 1.1.456 (Boston, MA, USA) or SPSS version 25.0 (Armonk, NY, USA). Continuous variables were presented as mean and standard deviation or median and interquartile ranges as appropriate. Groups were compared using Student *t*-test or Mann–Whitney *U* test for continuous variables and Chi-squared test or Fishers-exact test for categorical variables. Correction for age differences between groups was performed using a multivariable linear regression model. Inter- and intra-observer agreement was determined using weighted Cohen’s kappa. Missing values from CMR, echocardiography and Holter recordings were replaced using multiple imputations by chained equations based on all collected variables [18,19]. Cox proportional hazard models were used to analyze the relation between total Q-fQRS and the occurrence of SVA. SVA risk was described as hazard ratios (HR) with 95% confidence intervals (CI). A two-tailed p-value below 0.05 was considered statistically significant. 

## 3. Results

### 3.1. Study Population

The study population consisted of 336 individuals which included 221 (66%) patients with definite ACM (23% asymptomatic), 57 (17%) ACM-associated pathogenic variant carriers (81% asymptomatic) and 58 (17%) control subjects. Baseline characteristics are summarized in Table 1. Definite ACM patients (mean age 42.3 ± 14.6 years) were significantly older compared to pathogenic variant carriers (mean age 35.3 ± 16.3 years) and controls (mean age 27.4 ± 5.6) (overall group difference *p* < 0.001). Sex was not significantly different between groups (*p* = 0.811). The majority of ACM patients had a known pathogenic variant (76.5%), most commonly in *PKP2* (57%), *PLN* (16%) and *DSG2* (1%). By design, all pathogenic variant carriers had a pathogenic ACM-related variant with a similar gene distribution (*PKP2* 67%, *PLN* 23% and *DSG2* 11%). With regards to ECG parameters, the QTc interval was significantly prolonged in ACM patients compared to pathogenic variant carriers and controls (*p* < 0.001), whereas the QRS duration did not significantly differ between the groups (*p* = 0.066). Q-fQRS correlated with absolute TAD, another depolarization parameter from the TFC (*r* = 0.216, *p* = < 0.001, see Supplementary Figure S1). SVA before definite ACM diagnosis was present in 81 (37%) of definite ACM patients. At moment of definite ACM diagnosis, 88 (40%) ACM patients had an ICD implanted: 63 (72%) for primary prevention and 25 (28%) for secondary prevention of sudden cardiac death [17]. 

### 3.2. Feasibility and Reproducibility

Three independent investigators reviewed a random sample of ECGs (*n* = 30) while blinded for diagnosis and outcome. Assessment of total Q-fQRS was feasible and reproducible, with an interobserver agreement Kappa coefficient of 0.76–0.87 and an intraobserver agreement Kappa coefficient of 0.89–0.92.

### 3.3. Relation of Q-fQRS with Disease Status

**Figure 3:**
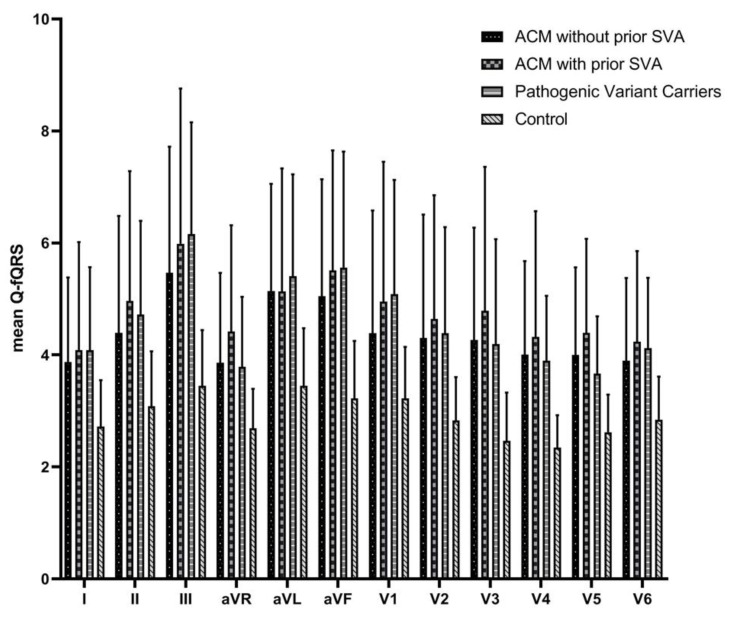
ACM = arrhythmogenic cardiomyopathy; ECG = electrocardiography; fQRS = fragmented QRS; SVA = sustained ventricular arrhythmia.

Total Q-fQRS is presented in Table 2 and Figure 3. Total Q-fQRS was significantly higher in ACM patients (54 ± 15) and pathogenic variant carriers (55 ± 10) compared to controls (35 ± 5) (overall group difference *p* < 0.001). In addition, pathogenic variant carriers had a significantly higher total Q-fQRS count compared to controls (55 ± 10 vs. 35 ± 5, *p* = 0.001), see Supplementary Figures S2–S4. Interestingly, ACM patients with prior SVA did not have a higher total Q-fQRS count compared to ACM patients without prior SVA (57 ± 20 vs. 53 ± 16, *p* = 0.081). Likewise, the total Q-fQRS count in pathogenic variant carriers was comparable to ACM patients with and without prior SVA (57 ± 20 vs. 55 ± 10, *p* = 0.883 and 53 ± 16 vs. 55 ± 10, *p* = 0.64, respectively). Age did not influence total Q-fQRS count when applying a multivariable linear regression model (*p* = 0.903, see Supplementary Table S5 and Supplementary Figure S6). Total Q- fQRS value did not differentiate symptomatic (*n* = 11) from asymptomatic (*n* = 46) pathogenic variant carriers (58 ± 3 vs. 54 ± 2, *p* = 0.271). However, pathogenic variant carriers with a major and a minor TFC (*n* = 34) had a significantly higher total Q-fQRS compared to those with only one major TFC (*n* = 23), (58 ± 10 vs. 51 ± 9, *p* = 0.015). By design, the major TFC were exclusively obtained by presence of a pathogenic variant and absence of any phenotypic sign of ACM at baseline. Furthermore, these pathogenic variant carriers without phenotypic ACM characteristics had a significantly higher total Q-fQRS compared to controls (51 ± 9 vs. 35 ± 5, *p* = 0.006). Pathogenic variant carriers who progressed to definite ACM diagnosis according to the TFC during follow up had a significantly higher total Q-fQRS compared to those pathogenic variant carriers who did not reach definite ACM diagnosis (60 ± 11 vs. 53 ± 9, *p* = 0.012).

### 3.4. Relation of Q-fQRS with Outcome

A total of 88 (40%) ACM patients experienced at least one episode of SVA during 9.8 ± 6.5 years of follow up. Appropriate ICD therapy (*n* = 43, 49%) and sustained VT (*n* = 44, 50%) were the most common SVA events, whereas SCA occurred only in one patient (1%). During follow-up, 68 definite ACM patients and two pathogenic variant carriers received an ICD. Both pathogenic variant carriers (one carrier of a *PKP2* variant and one carrier of a *PLN* variant) were included in the study when they did not fulfil the 2010 TFC. During follow up, both carriers were diagnosed with ACM according to the TFC based on progression of ECG abnormalities, PVC burden on Holter recording and CMR abnormalities. Implantation of an ICD was deemed appropriate by their treating physicians [17]. However, SVA did not occur in the pathogenic variant carriers group during 8 ± 5 years of follow up. Age, sex, symptoms and right ventricular ejection fraction (RVEF) were significantly associated with SVA events in patients with ACM without prior SVA. However, in univariable analysis Q-fQRS (0.99 (0.96–1.02), *p* = 0.335) was not related to SVA episodes during follow up (Table 3).

### 3.5. Relation of Q-fQRS with Genotype

**Figure 4:**
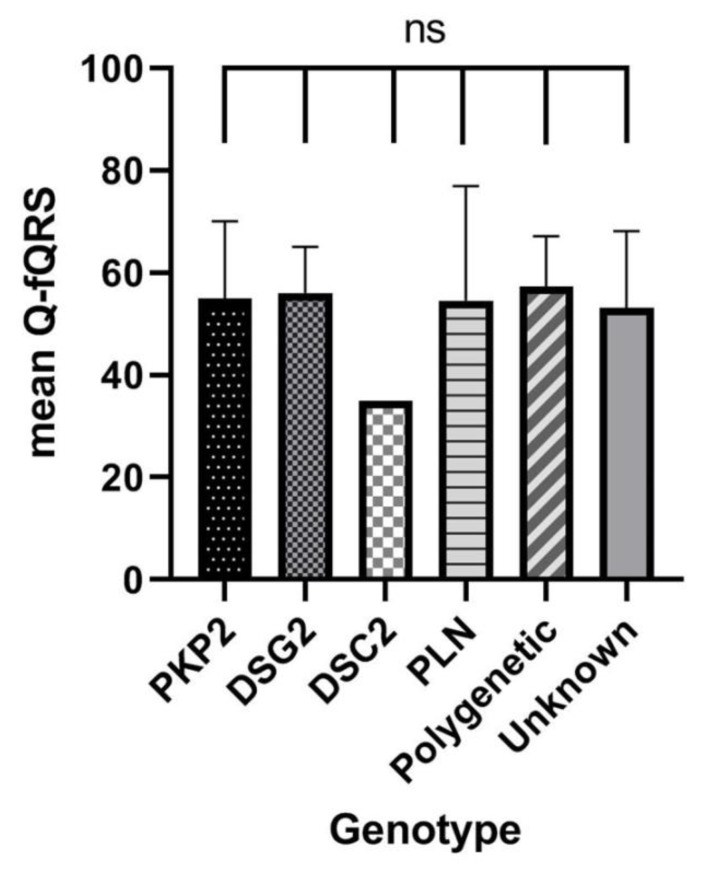
*PKP2* = Plakophilin-2; *DSG2* = Desmoglein-2; *DSC2* = Desmocollin-2; *PLN* = Phospholamban; Polygenetic = multiple pathogenic ACM-related variants, Unknown = no pathogenic variant identified using genetic screening.

The Q-fQRS count did not significantly differ between presence or absence of specific pathogenic variants in both definite ACM patients and pathogenic variant carriers (*p* = 0.817, Figure 4). Age, sex, symptoms and RVEF were significantly associated with SVA in genotype positive ACM patients without prior SVA. However, Q-fQRS count was not related to the occurrence of SVA (HR 0.99 (0.96–1.03), *p* = 0.693, Table 4).

## 4. Discussion

Fragmented QRS complexes are a surface ECG manifestation of prolonged activation pathways and conduction slowing which give rise to activation delay [3,5,6,7]. In ACM, local activation delay is integrated in the diagnostic 2010 TFC by recording of late potentials, prolonged TAD, and epsilon waves [2]. Although not used in the current TFC, fQRS is a comparable depolarization parameter. Qualitative fQRS assessment is hampered by methodological drawbacks which give rise to poor reproducibility [10,12]. To overcome these drawbacks and to avoid qualitative operator-dependent interpretation, several adaptations in fQRS assessment have been proposed such as inclusion of morphologic criteria [12] and automated quantification [20]. However, these modifications were not specifically proposed for ACM cohorts. 

Our study has several interesting findings. First of all, the new standardized quantitative method used for Q-fQRS count appeared to be feasible with acceptable intra- and inter-observer agreement. Second, compared to a control group, we observed a significantly increased amount of total Q-fQRS count in definite ACM patients without prior SVA. Third, both in symptomatic and asymptomatic pathogenic variant carriers who did not fulfil the TFC for ACM diagnosis, a similar elevated total Q-fQRS count was found. Even pathogenic variant carriers without any phenotypic signs of ACM showed significant elevation of Q-fQRS compared to controls. Apparently and most importantly, increase of Q-fQRS occurs early in the ACM disease process, suggesting its potential diagnostic value in the concealed stage. Fourth, pathogenic variant carriers with progression to definite ACM during follow up, were associated with a higher Q-fQRS at baseline. However, an association between total Q-fQRS and SVA was absent in both definite ACM patients and pathogenic variant carriers. 

Previous studies assessed fQRS in patients with ACM as a binary phenomenon, whereas in reality it is more likely to resemble a spectrum of altered activation pathways and pro-arrhythmic substrates [8,9]. Based on literature and current pathophysiological concepts of re-entry related ventricular arrhythmia, one would expect the lowest Q-fQRS in asymptomatic pathogenic variant carriers and the highest Q-fQRS count in ACM patients with a high SVA burden. Unfortunately, we were unable to study the relation between scar burden and Q-fQRS because late gadolinium enhancement imaging with CMR was not routinely performed for all ACM patients diagnosed between 1991 and 2012. Using the binary qualitative analysis Peters et al. reported an fQRS incidence of 83%, whereas Canpolat et al. reported fQRS in 59% of ACM patients [8,9]. In our study, the Q-fQRS was comparable between ACM patients with or without prior SVA, in line with observations of Peters et al. [9]. Sub-analysis for ACM patients with or without prior SVA was not performed in previous studies [8,9]. In the present study, pathogenic variants were identified in 77% of the definite ACM patients and pathogenic variants were labeled as a major criterion according the 2010 TFC [2]. Thus, less phenotypic TFC were required for ACM diagnosis fulfilment in our study compared to prior studies which did not report molecular-genetic data [8,9]. This is illustrated by the overall normal RVEF (mean RVEF value: 48%) of definite ACM patients in the present study compared to the lower mean RVEF (39.5%) in the study of Canpolat et al. 

The ECG settings in our study were chosen according to the current consensus in literature to use a low pass filter of 150 Hz [4]. Fast deflections in the QRS complex might be underestimated when using a lower cutoff as low pass filter (<150 Hz), as subtle fragmentations in the QRS complex are not identified. Previous studies used different low pass filter settings (40–50 Hz) that most likely have influenced fQRS detection [8,9]. In previous studies, use of AAD was not reported [9] or ECGs were recorded while a substantial number of patients were on either amiodarone (19%) or sotalol (14%) [8]. The effect of AAD on QRS fragmentation is not well known, but due to decreasing myocardial conduction velocity the amount of Q-fQRS might further increase. To deal with this potential effect, ECGs of patients on AAD during ECG acquisition were excluded in the present study. Although our quantification method of fragmented QRS complexes is feasible with acceptable intra- and interobserver agreement, future studies using this method are needed to confirm reproducibility. In addition, future studies in ACM patients should focus on the development of automated fQRS quantification software to further improve user-independency, similar as already reported for other patient categories [20].

Interestingly, Q-fQRS was similar in ACM patients and asymptomatic pathogenic variant carriers without ACM fulfilment and significantly lower in control subjects. This indicates that Q-fQRS is an early sign of disease penetrance due to electrical dyssynchrony or alteration of activation pathways. These findings are in line with earlier observations of electrical changes of the myocardium preceding structural changes in patients with ACM and carriers of ACM-associated pathogenic variants [21,22]. Moreover, monitoring of Q-fQRS might be a promising, low-cost and non-invasive marker to aid early detection of disease progression in carriers of pathogenic ACM-associated variants. 

The population characteristics of the subgroups were not balanced due to the retrospective nature of this study, which compromises the generalizability of the results. A limitation that may affect interpretation of our results is the age difference between the groups of definite ACM patients, pathogenic variant carriers and controls. The significantly younger pathogenic variant carrying group had a similar total Q-fQRS count compared to the definite ACM group. However, the age difference between the pathogenic variant carriers group and the control group did not influence total Q-fQRS when applying a multivariable linear regression model, showing a very poor and insignificant relation between age and fQRS (*p* = 0.903). Furthermore, performing age-sex matching between pathogenic variant carriers and controls did not change our findings, see Supplementary Figure S5. The absence of a relation between Q-fQRS and SVA in our study may be explained by adrenergic mediated triggering of SVA besides scar related re-entry as the mechanism of SVA in ACM [23,24]. Furthermore, changes in clinical heart failure management, arrhythmia management, and genetic family screening protocols could have affected SVA incidence during the long-lasting inclusion period. No correction for treatment effects of drug therapy for heart failure, AAD therapy or VT ablation after study inclusion was applied. Appropriate ICD therapy episodes were included as SVA episodes, although some episodes might have been terminated spontaneously without requiring ICD therapy. Furthermore, ICD algorithms improved during the inclusion period, which could have lowered ICD therapy incidence compared to previous studies.

## 5. Conclusions

Both definite ACM patients and pathogenic variant carriers not fulfilling ACM diagnosis have a higher Q-fQRS than control subjects. This may indicate that increased Q-fQRS is an early sign of disease penetrance, suggesting its potential role in diagnosis of early stage ACM. In concealed and early stages of ACM the role of Q-fQRS in risk stratification is limited.

## Figures and Tables

**Table 1:** Baseline characteristics.

	Overall (*n* = 336)	Definite ACM (*n* = 221)	Carriers (*n* = 57)	Control (*n* = 58)	*p* Value ^#^
Demographics					
Age (years.)	39 ± 15	42 ± 15	35 ± 16 *	27 ± 6	<0.001
Sex (male)	181 (54)	119 (54)	29 (51)	33 (57)	0.811
Symptoms	182 (54)	171 (77)	11 (19) *	0 (0)	<0.001
Cardiac syncope	52 (16)	52 (24)	0 (0)	0 (0)	<0.001
Genetics					
Pathogenic variant	226 (67)	169 (77)	57 (100)	NA	<0.001
*PKP2*	163 (49)	125 (57)	38 (67)	NA	<0.001
*DSG2*	9 (3)	3 (1)	6 (11)	NA	<0.001
*PLN*	49 (15)	36 (16)	13 (23)	NA	<0.001
ECG					
PR interval (ms)	157 ± 27	161 ± 28	145 ± 20	154 ± 24	<0.001
QRS duration (ms)	96 ± 15	97 ± 17	92 ± 12	97 ± 10	0.066
QTc interval (ms)	419 ± 26	425 ± 28	413 ± 24	405 ± 14	<0.001
TWI V1–2	136 (41)	127 (58)	8 (14) *	1 (2)	<0.001
TWI V1–3	102 (30)	102 (46)	0 (0)	0 (0)	<0.001
TWI V4–6	18 (5)	18 (8)	0 (0)	0 (0)	0.007
TAD (ms)	56 ± 10	56 ± 16	52 ± 14	46 ± 9	<0.001
Arrhythmia					
PVC count/24 h on Holter	851 (113–2623)	1076 (534–3403)	20 (2–492)	NA	<0.001
NSVT	111 (33)	101 (46)	10 (18)	NA	<0.001
SVA at baseline	81 (24)	81 (37)	0 (0)	NA	<0.001
Imaging					
RVEF (%)	48 (45–53)	48 (41–48)	53 (49–59)	53 (49–56)	<0.001
LVEF (%)	59 (53–62)	62 (52–62)	58 (53–60)	58 (54–63)	0.255
RV Volume (mL/m^2^)	100 (90–118)	100 (90–118)	93 (83–107)	102 (93–120)	0.001

ACM = arrhythmogenic cardiomyopathy; PKP2 = plakophilin-2; DSG2 = desmoglein-2; PLn = phospholamban; TWI = T wave inversion; TAD = terminal activation duration; PVC = premature ventricular complex; NSVT = non-sustained ventricular tachycardia; SVA = sustained ventricular arrhythmia; RVEF = right ventricular ejection fraction; LVEF = left ventricular ejection fraction; RV = right ventricle. # Overall group difference. * Significant difference between pathogenic variant carriers with definite ACM diagnosis and controls.

**Table 2:** Fragmentation of the QRS complex.

Parameters	ACM with Prior SVA (*n* = 81)	ACM without Prior SVA (*n* = 140)	Pathogenic Variant Carriers (*n* = 57)	Control (*n* = 58)	*p* Value
Q-fQRS anterior leads (V1–4)	19 ± 8	17 ± 6	18 ± 4	11 ± 2	<0.001
Q-fQRS inferior leads (II, III, aVF)	16 ± 7	15 ± 6	16 ± 5	10 ± 3	<0.001
Q–fQRS lateral leads (I, aVL, V5–6)	18 ± 6	17 ± 5	17 ± 3	12 ± 2	<0.001
Q-fQRS lead aVR	4 ± 2	4 ± 2	4 ± 1	3 ± 1	<0.001
Total Q-fQRS	57 ± 20	53 ± 16	55 ± 10	35 ± 5	<0.001

ACM = arrhythmogenic cardiomyopathy; SVA = sustained ventricular arrhythmia; Q-fQRS = quantitative fQRS. Significant differences in total Q-fQRS: pathogenic variant carriers vs. controls *p* = 0.0001, ACM patients with prior SVA vs. controls *p* = 0.0001, ACM patients without prior SVA vs. controls *p* = 0.0001. For detailed comparisons see Supplementary Figure S4.

**Table 3:** Univariable and multivariate analysis of SVA risk in definite ACM patients without prior SVA.

Univariable			Multivariable		
	Hazard Ratio (95% CI)	*p* Value		Hazard Ratio (95% CI)	*p* Value
Age	0.98 (0.96–1.00)	0.071	Age	0.96 (0.93–0.99)	0.008
Sex	2.19 (1.08–4.44)	0.030	Sex	2.01 (0.92–4.41)	0.080
Symptoms	3.05 (1.17–7.93)	0.022	Symptoms	4.91 (1.43–16.84)	0.011
PR interval	0.99 (0.98–1.01)	0.206			
QRS duration	0.99 (0.97–1.02)	0.529			
QTc interval	1.00 (0.99–1.01)	0.856			
RVEF	0.96 (0.92–0.99)	0.013	RVEF	0.97 (0.93–1.00)	0.064
LVEF	0.99 (0.94–1.03)	0.536			
Total Q-fQRS	0.99 (0.96–1.02)	0.335			

RVEF = right ventricular ejection fraction; LVEF = left ventricular ejection fraction; Q-fQRS = quantitative fragmented QRS count.

**Table 4:** Univariable and multivariable analysis of SVA risk in genotype positive definite ACM patients without prior SVA.

Univariable			Multivariable		
	Hazard Ratio (95% CI)	*p* Value		Hazard Ratio (95% CI)	*p* Value
Age	0.99 (0.96–1.02)	0.591	Age	0.96 (0.92–0.99)	0.044
Sex	2.32 (1.01–5.30)	0.046	Sex	3.71 (1.25–11.03)	0.018
Symptoms	3.03 (1.03–8.93)	0.044	Symptoms	4.93 (1.05–23.05)	0.042
PR interval	0.99 (0.98–1.01)	0.476			
QRS duration	0.98 (0.95–1.01)	0.312			
QTc interval	1.00 (0.99–1.01)	0.897			
RVEF	0.93 (0.89–0.97)	0.002	RVEF	0.93 (0.88–0.98)	0.010
LVEF	0.98 (0.93–1.03)	0.372			
Total Q-fQRS	0.99 (0.96–1.03)	0.693			

RVEF = right ventricular ejection fraction; LVEF = left ventricular ejection fraction; Q-fQRS = quantitative fragmented QRS count.

## Supplementary Materials

The following are available online at https://www.mdpi.com/2077-0383/9/2/545/s1, Figure S1: Correlation between terminal activation duration and Q-fQRS, Figure S2: Q-fQRS count according to Phenotype, Figure S3: Receiver operating characteristics (ROC) curve of Q-fQRS, Figure S4: Differences between Q-fQRS according to phenotype and ECG leads, Figure S5: Boxplots of Q-fQRS after age-sex matching between pathogenic variant carriers and control subjects, Figure S6: Correlation between age and Q-fQRS according to phenotype, Table S1: Correction using multivariable linear regression for age differences between groups.
