# Supplementary material for: Quantitative Approach to Fragmented QRS in Arrhythmogenic Cardiomyopathy: From Disease towards Asymptomatic Carriers of Pathogenic Variants
Source: J Clin Med. 2020 Feb 17;9(2):545. doi: 10.3390/jcm9020545 (PMC7073517; doi:10.3390/jcm9020545)
Supplement: Supplementary file 1 [file jcm-09-00545-s001.pdf]

Supplementary material

Supplementary Table 1. Correction using multivariate linear regression for age difference between groups.

	Unstandardized B	Coefficients Std. Error	Standardized Coefficients Beta	t	P value
Age	0.007	0.60	0.007	0.123	0.903
ACM without prior SVA	17.588	2.507	0.519	7.014	<0.001
ACM with prior SVA	22.377	2.717	0.574	8.235	<0.001
Pathogenic variant carriers	20.064	2.830	0.451	7.091	<0.001

Legend Table 1: ACM = arrhythmogenic cardiomyopathy; Q-fQRS = mean quantitative fragmented QRS count.

Supplementary Figure 1. Correlation between Terminal Activation Duration and Q-fQRS.

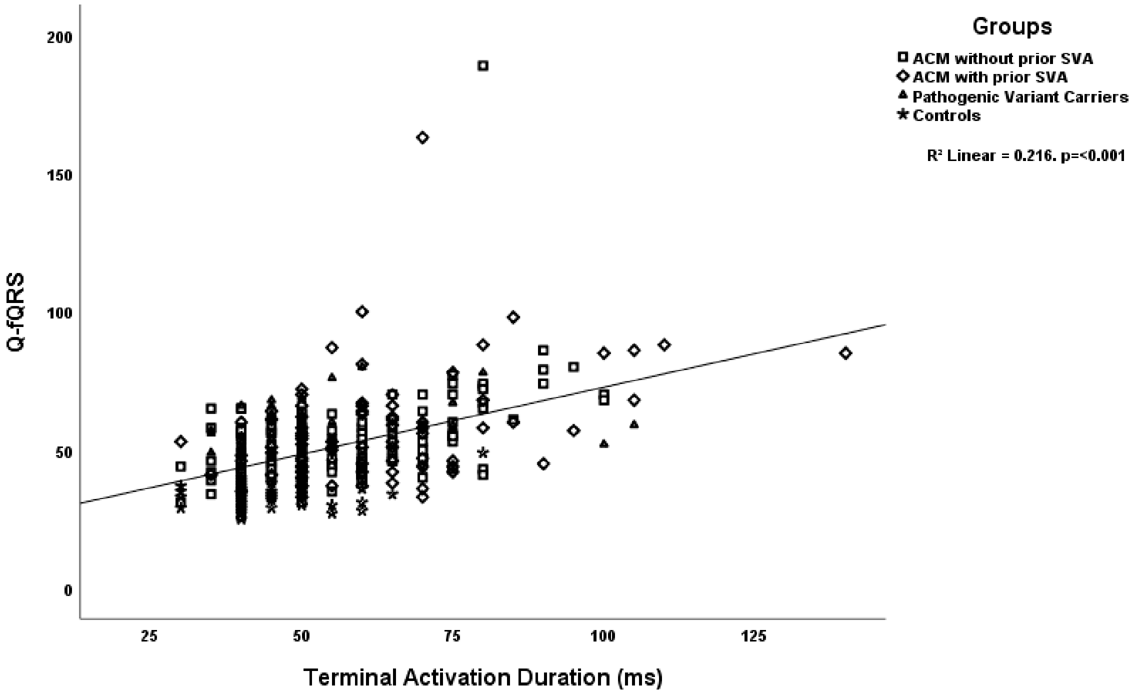

Legend supplementary Figure 1: Q-fQRS = mean quantitative fragmented QRS count.

Supplementary figure 2. Q-fQRS count according to Phenotype.

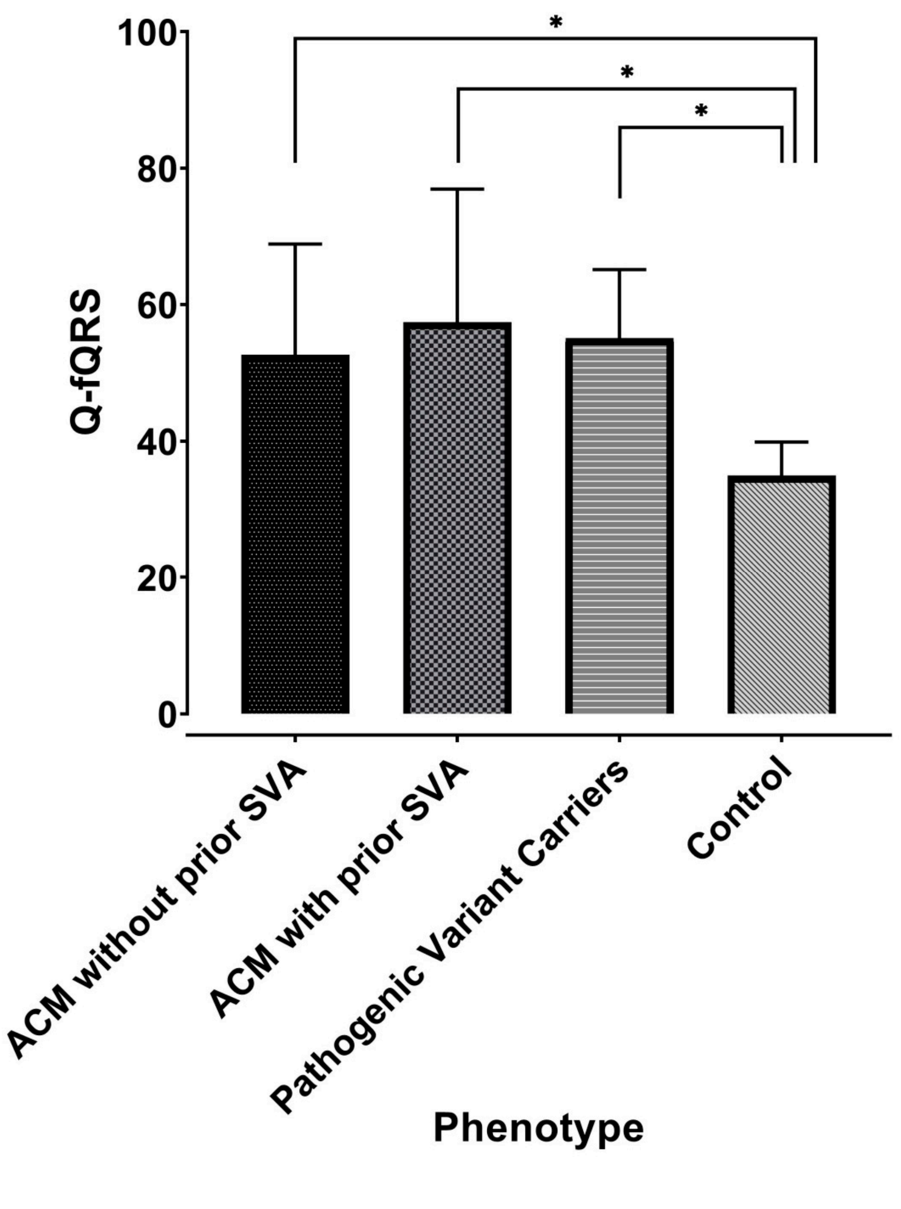

Legend supplementary figure 2: ACM = arrhythmogenic cardiomyopathy; Q-fQRS = mean quantitative fragmented QRS count. Differences in Q-fQRS: ACM patients without prior SVA vs. ACM patients with previous SVA p=0.081, ACM patients with prior SVA vs. pathogenic variant carriers p=0.883, pathogenic variant carriers vs. controls p=0.0001, ACM patients

without prior SVA vs. pathogenic variant carriers p=0.64, ACM patients with prior SVA vs. controls p=0.0001, ACM patients without prior SVA vs. controls p=0.0001.

Supplementary Figure 3. Receiver operating characteristics (ROC) curve Q-fQRS.

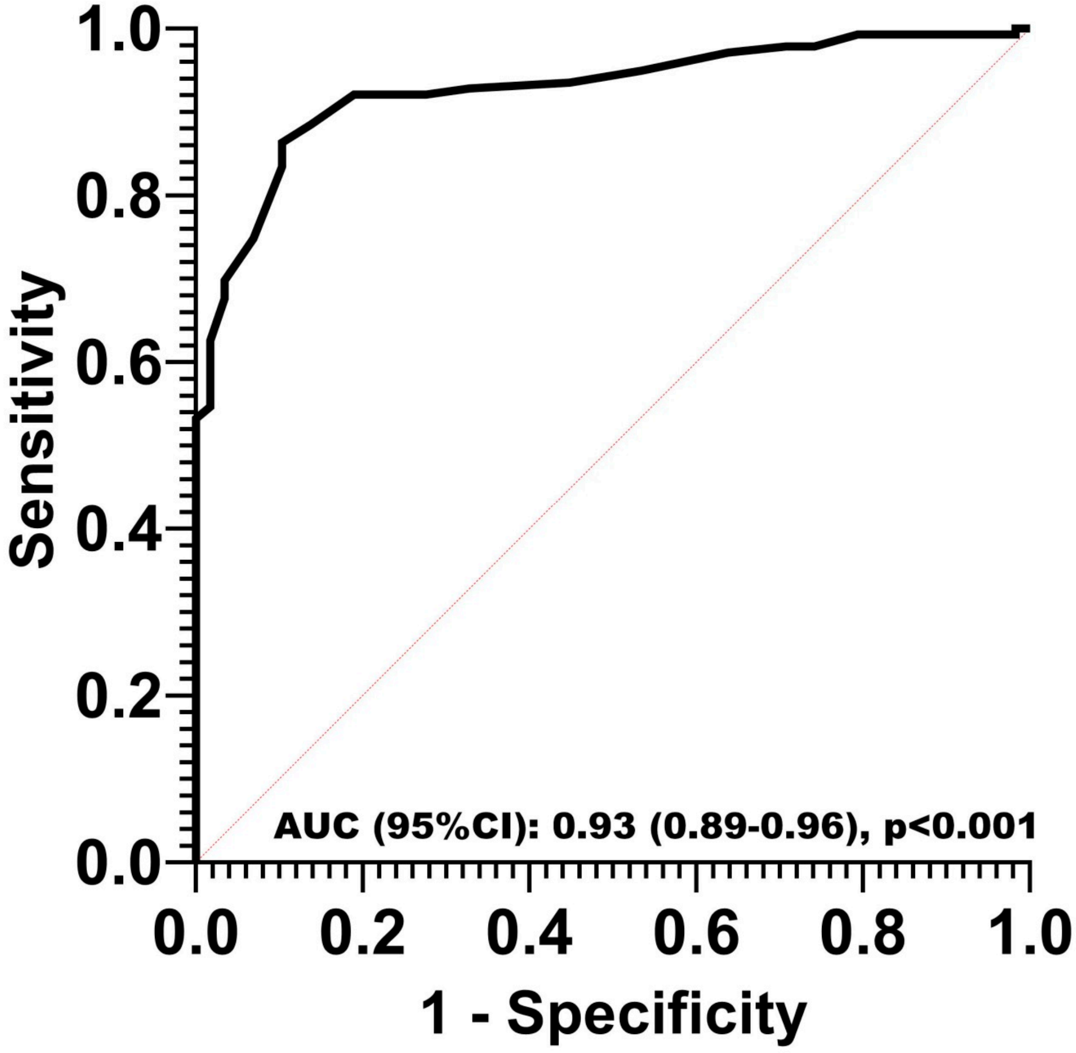

Legend supplementary Figure 3: receiver operator characteristics (ROC) curve for Q-fQRS as predictor of definite ACM diagnosis.

Supplementary Figure 4. Differences between Q-fQRS per Phenotype and ECG leads.

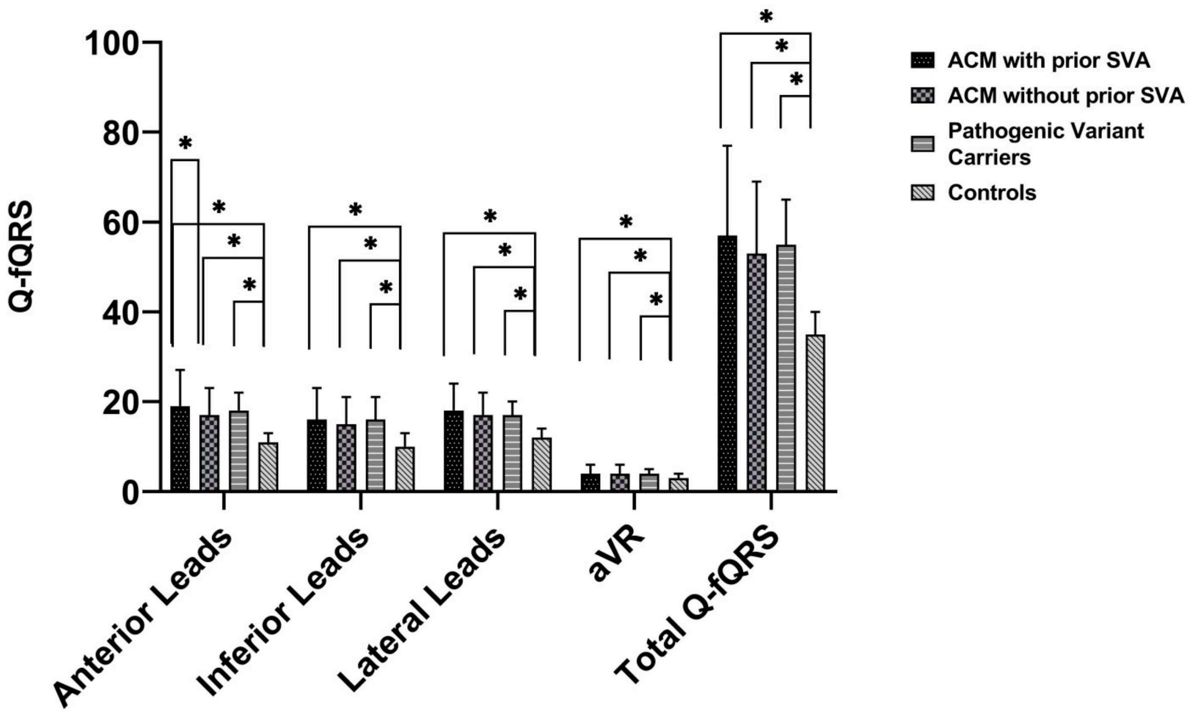

Legend supplementary Figure 4: ACM = arrhythmogenic cardiomyopathy; SVA = sustained ventricular arrhythmia; Q-fQRS = mean quantitative fragmented QRS count. * significant difference between groups.

Supplementary Figure 5. Boxplots of Q-fQRS after age and sex matching betweem pathogenic variant carriers and control subjects.

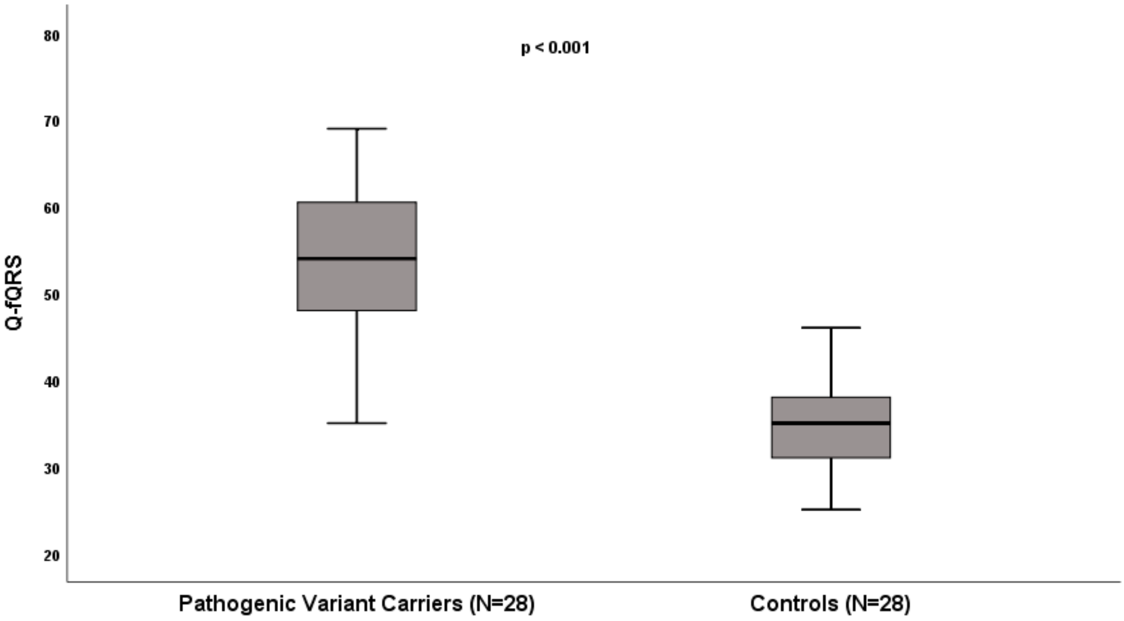

Legend supplementary Figure 5: Q-fQRS = mean quantitative fragmented QRS count.

Supplementary Figure 6. Correlation age and Q-fQRS according to phenotype.

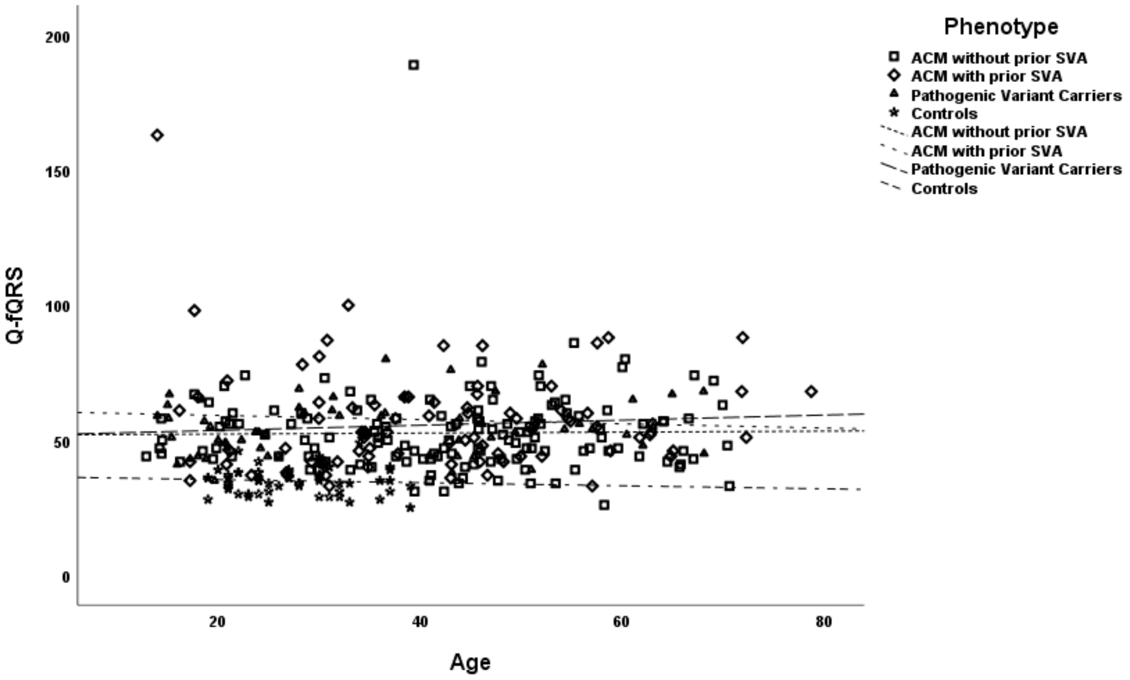

Legend supplementary Figure 6: Q-fQRS = mean quantitative fragmented QRS count; ACM without prior SVA = R^2 Linear: < 0.001, p = 0.851; ACM with prior SVA: R^2 Linear = 0.004, p = 0.599; Pathogenic Variant Carriers: R^2 Linear = 0.024, p = 0.254; Controls: R^2 Linear = 0.004, p = 0.628.
